# Therapeutic Note on Chloral

**Published:** 1870-06

**Authors:** J. Y. Simpson


### Miscellaneous.

### Therapeutic Note on Chloral.

BY SIR J. Y. SIMPSON.

Sir J. Y. Simpson gives (*Med. Times and Gazette*, Jan. 1, 1870) a very interesting account of his experience with chloral. He thinks that this remedy will prove of immense value in the practice of medicine, surgery, and midwifery.

"Hitherto," he states, "I have principally employed it as an hypnotic and anodyne. In sufficient doses I have found it, as a general law, as sure a producer ot sleep and soother of pain as opium or any of its preparations. It is usually swifter in the induction of its narcotism, more tranquil in its action, and more prolonged in its effects than opiates are when taken as hypnotics; but above all, it seems in a great measure, free from some of the minor drawbacks and disagreeable accompaniments produced by a full and large dose of opium. In this respect it appears to me to fulfil successfully the indications which I predicted in the extract above given, of being a narcotic as powerful, and indeed more powerful, than opium, 'yet without either its direct constipating effects, or its indirect tendency to excite subsequent nausea, vomiting, etc.' The sleep induced by a full dose of it steals on without any premonitory symptoms. It is usually deeper, and yet more quiet and calm, than that produced by opium; and it does not leave subsequently the thirst, dry throat and tongue, disturbance of stomach and appetite, and languor of mind as well as body, which most persons unaccustomed to the use of opium commonly feel after a deep and narcotic dose of that drug.

"Ever and anon cases are well known to occur in practice in which patients declare their inability to take opium in any form without suffering severely from nausea, faintness, restlessness, and other evil effects. In several such cases I have now used chloral as an hypnotic with perfect success. A patient here at present from New York assures me that the preparations of opium and other vegetable anodynes have always acted upon her as poisons, and without producing their usual hypnotic effects. 'Such,' she writes me, 'being my experience of anodynes, I was unwilling, as you remember, to take chloral, and hoped nothing whatever from it. It was administered to me in two half-doses, [thirty grains each ;] the first dose, taken in the daytime, with light in the room and my people walking about, did not put me to sleep, but it soothed and calmed me completely. The second dose, given at night, was followed by nearly four hours of natural and refreshing sleep. I felt neither giddiness nor heaviness on waking, and neither then nor later did I experience any sensation of nausea as after other anodynes.'

"Two or three weeks ago I had under my care an old patient, a lady of great sensitiveness and intellectual power, from one of the midland counties of England. When last in Edinburgh she was the subject of a slight operation, and twice took a dose of chloral at night to induce rest. She slept under it quietly and refreshingly, far beyond her usual breakfast hour. Opium, henbane, and other anodynes had, when used, generally induced in her disturbed sleep, occasional sleep-talking, and sometimes somnambulism. A few days before coming on this last occasion to Edinburgh, she had a conversation with her mother regarding the kind of monument which they should erect over the grave of her father, who died two or three months since. That same day she had traveled up from Wales, felt ill, and had given to her a dose of henbane towards bedtime, with the hope of producing rest. In the middle of the night her husband was awoke by the ringing of his door bell, a shower of small stones launched against his bedroom window, and the dog barking within. On rushing down and opening the door he found his wife, whom he believed to be in bed, standing outside. The henbane draught had produced a fit of sleep-walking. After her husband and she had fallen asleep she had risen, dressed herself in her day clothes over her night clothes, removing for that purpose her bonnet and muff out of their special receptacles, and, in the middle of a dark and wet night, had walked off to a distance of two miles. She awoke with her left hand holding her two gloves within her muff, and the right hand grasping the cold iron handle of the inner gate of the churchyard in which her father had recently been buried. After using the chloral she expressed to me great satisfaction at the idea that she had now a medicine which seemed to produce nothing but a tranquil sleep, quite different from the disturbing effects of the narcotics which she had previously taken; and two days ago I saw an order from England at the apothecary's for several doses to be forwarded to her.

"Sometimes chloral produces its hypnotic effects when opium, from its long-continued use, had ceased to do so. To a patient who has had daily morphia injected subcutaneously for some years for neuralgia of the side under the hands of different practitioners, my assistant, Dr. Bell, gave at my request a drachm dose of chloral. Latterly a grain of morphia has been injected daily with the effect of relieving the pain, but without producing sleep. She swallowed the dose of chloral early in the afternoon, and was asked to lie down in bed. I saw her in a quarter of an hour afterwards deeply asleep; and the lifting of one eyelid to look at the dilated pupil did not awaken her. She awoke out of the slumber free from her neuralgia.

"I am not aware of any special contra-indications to the employment of chloral when used for somniferous purposes. Even in head and chest affections, where I should have been chary of having recourse to opium as an hypnotic, I have employed chloral with perfect success. The contra-indications to opium offered by a tendency to constipation, etc., do not exist against chloral.

"Like all other remedies in the pharmacopœia, it will no doubt occasionally fail to produce its desired effect; but as seldom so, perhaps, as most of them. In a few instances the sleep induced by it has been dreamy and hysterical, particularly when the patient was not kept in a state of perfect quietude; but these are rare exceptions to the general rule.

"In the present remarks I have spoken specially of the somniferous or hypnotic powers of chloral. I have used it for other purposes, but it is not my intention to dwell upon them at present. It will not fulfill all the many and almost endless indications for which opium is used in medicine; but I have seen enough to convince me that it will prove a very valuable anodyne in some cases of neuralgia, hysteralgia, dysmenorrhœa, pleurodynia, etc., and in the pains attendant upon cancer and acute local inflammations. In some cases of irritable bladder and chronic cystitis I have found it give the patient much longer and more perfect rest than large doses of opium. In several instances it has seemed to me, when given in small and repeated doses, to soothe down both acute and chronic cough with remarkable effect; and I have known it to relieve asthma. Lately in a young lady whom I saw, in consultation with Dr. Taylor, suffering under a severe attack of congestive bronchitis with some hæmoptysis, and orthopnœa, a small dose (twenty grains) of chloral was given at night. 'She speedily fell asleep,,' wrote Dr. Taylor to me the next day, 'and slept soundly until 4 A. M., when she sat forward in bed and coughed, but appeared to be only half awake. When I called in the morning at 10.30 she was still enjoying a most placid slumber. 'As I contrast,' Dr. Taylor adds, 'the distressed and audible breathing of last night with the tranquil sleep and improved state of the patient to-day, I cannot help concluding that chloral has a directly sedative effect on the whole respiratory surfaces.'

"Occasionally I have exhibited chloral in continuous small doses for one, two, or more weeks in succession, and apparently with most marked benefit, particularly in cases of chorea, threatened or incipient insanity, etc. A patient from Illinois, who, for several years, has always regularly suffered excruciating spasmodic pain in the left iliac region, attended with some discharge, for eight days before menstruation began (she has disease of the fundus uteri and left Fallopian tube,) has, during the last two periods, kept at bay this old and formidable suffering by taking chloral night and morning during the threatenings of it. She strongly assures me that formerly she had used very large doses of opium and other anodynes without any such favorable effect. I have found the parturient uterus to go on contracting regularly and strongly when the patient was so deeply asleep under chloral as to be only imperfectly wakened up with the expulsive effort of labor.

"The dose of chloral to an adult for an hypnotic which I have usually employed has varied from 50 to 60 grains; but 25 to 30 grains suffice in some patients. In a case of long-standing sleeplessness, and which has resisted great doses of opium, Indian hemp, etc., etc., 120 grains failed to produce any effect. When used for anodyne and other medicinal purposes, a continuation of smaller doses—as 10 or 20 grains several times a day—is sufficient.

"In administering chloral I have given it only by the mouth and by enema; almost always as a draught. It is somewhat acrid and pungent to most palates, and hence requires to be diluted well with water, and to have added to it a large quantity of syrup."—*Medical News.—St. Louis Medical Journal.*

